# Understanding the Role of Pattern Geometry on Nanofiltration Threshold Flux

**DOI:** 10.3390/membranes10120445

**Published:** 2020-12-21

**Authors:** Anna Malakian, Zuo Zhou, Lucas Messick, Tara N. Spitzer, David A. Ladner, Scott M. Husson

**Affiliations:** 1 Department of Chemical and Biomolecular Engineering, Clemson University, Clemson, SC 29634, USA; amalaki@clemson.edu (A.M.); lmessic@clemson.edu (L.M.); tspitze@clemson.edu (T.N.S.); 2 Department of Environmental Engineering and Earth Sciences, Clemson University, Clemson, SC 29634, USA; zuoz@clemson.edu (Z.Z.); ladner@clemson.edu (D.A.L.)

**Keywords:** colloidal fouling, membrane patterning, membrane surface modification, threshold flux, thin-film composite membranes

## Abstract

Colloidal fouling can be mitigated by membrane surface patterning. This contribution identifies the effect of different pattern geometries on fouling behavior. Nanoscale line-and-groove patterns with different feature sizes were applied by thermal embossing on commercial nanofiltration membranes. Threshold flux values of as-received, pressed, and patterned membranes were determined using constant flux, cross-flow filtration experiments. A previously derived combined intermediate pore blocking and cake filtration model was applied to the experimental data to determine threshold flux values. The threshold fluxes of all patterned membranes were higher than the as-received and pressed membranes. The pattern fraction ratio (PFR), defined as the quotient of line width and groove width, was used to analyze the relationship between threshold flux and pattern geometry quantitatively. Experimental work combined with computational fluid dynamics simulations showed that increasing the PFR leads to higher threshold flux. As the PFR increases, the percentage of vortex-forming area within the pattern grooves increases, and vortex-induced shielding increases. This study suggests that the PFR should be higher than 1 to produce patterned membranes with maximal threshold flux values. Knowledge generated in this study can be applied to other feature types to design patterned membranes for improved control over colloidal fouling.

## 1. Introduction

Colloidal fouling is an impediment to pressure-driven membrane operations. For nanofiltration (NF) membranes, fouling can occur by accumulation of particles such as colloidal silica, iron, aluminum, manganese oxides, and calcium carbonate precipitates on the membrane surface [1]. This foulant layer introduces an additional hydraulic resistance that reduces water permeability [2]. There have been systematic studies on reverse osmosis (RO) and NF colloidal fouling that examined the physical and chemical aspects of particle-membrane interactions and the application of antifouling functional groups on membranes surfaces [3,4,5,6,7]. Chemical modification is the most common method to decrease fouling [8,9,10,11]. While somewhat effective for this purpose, chemical modification can negatively impact other membrane performance metrics such as flux and selectivity [12].

Physical modification of membrane surfaces attempts to mitigate the fouling by decreasing the random roughness [3,13]. Introduction of ordered geometric or biomimetic patterns on membrane surfaces decreases fouling by influencing the hydrodynamics at the solid–liquid interface [14,15,16,17,18]. Different research groups have investigated different pattern shapes and sizes. Lee et al. [19] and Won et al. [20] both designed microscale prism patterns. Jang et al. [21] studied both nanometer-and micrometer-scale patterns achieved through nanoimprint lithography. Zhou et al. [22] investigated several line-and-groove patterns, rectangular and circular pillars, and pyramids within the nanometer- to micrometer-scale range. Ling et al. [23] studied microscale pillars on RO membranes. Irregular shapes such as sharkskin mimetic patterns also have been investigated for improving biofouling resistance [24].

Recently, we demonstrated that the introduction of a nanoscale line-and-groove pattern on NF membranes could increase their threshold flux by 20–30% during filtration of colloidal suspensions [25]. Using atomic force microscopy of particle-fouled membranes, we observed below threshold flux that particles aligned along the pattern grooves in a way that appears to be consistent with the mechanism of selective deposition proposed by ElSherbiny et al. [26]. According to this mechanism, particles accumulate selectively in the pattern valleys due to lower shear stress. In our case, these low-shear regions occur within the pattern grooves, which delays cake layer formation and leads to higher values of threshold flux. Based on that work, we theorized that the pattern geometry would affect the threshold flux by altering the shear stress profiles at the interface, as we have seen in computational simulation work [22].

Computational simulations contribute to the analysis of transport phenomena adjacent to membrane surfaces, as the details of fluid flow within a membrane device can be difficult to measure experimentally. In the simulations, the Navier–Stokes, continuity, and convection–diffusion equations are fully coupled to describe fluid flow and mass transfer within a membrane channel [27,28]. Several research groups, ours included, have conducted computational fluid dynamics (CFD) simulations on patterned membranes, trying to understand their mechanisms for fouling control. One hypothesis is that increased turbulence at the apex of the pattern surface could lead to reduced deposition of foulants [15,20]. Others have posited that higher shear stress at the apex region efficiently reduces the attachment of the particles, keeping them away from the fouling region [19,23,29].

In this study, we investigated the influence of pattern geometry on threshold flux. We formed nanoscale line-and-groove patterns with different spacing dimensions on NF membranes through thermal embossing. Threshold flux was determined by applying a combined intermediate pore blocking and cake filtration model to the experimental data, as described previously [25,30]. CFD simulations were performed to analyze the velocity streamlines and shear stress profiles adjacent to the patterned membrane surfaces.

## 2. Materials and Methods

### 2.1. Materials

Ammonium hydroxide solution (ACS grade, 28.0–30.0% NH_3_ basis), ethyl alcohol (anhydrous, 200 proof), N,N-dimethylformamide (DMF, 99.8%), succinic anhydride (99%), and tetraethyl orthosilicate (TEOS, 99.99%) were purchased from MilliporeSigma (St. Louis, MO, USA) and used as received. Polyamide thin-film NF membrane sheets (GE HL) were purchased from Sterlitech Corp. (Kent, WA, USA). The silicon line-and-groove stamps were purchased from Digi-Key Electronics (Thief River Falls, MN, USA). Deionized (DI) water was used to prepare the solutions.

### 2.2. Membrane Patterning

Membranes were patterned using thermal embossing. Membrane coupons (1.50 cm × 4.25 cm) were cut from membrane sheets. Silicon stamps with different feature sizes (Table 1) were used to pattern the membranes in a Carver press (model 3851-0, Wabash, IN, USA). The temperature of the hot press plates was set to 65 °C. A stamp was placed on top of the membrane with its polyamide selective layer facing upward, and the sample was sandwiched between two pieces of thermally conductive silicone rubber to distribute the force evenly across the membrane. The press plates were closed until the set pressure was reached. Embossing was conducted for 15 min with an applied pressure of 3.55 MPa. For “pressed” membranes, the same procedure was applied using a flat silicon wafer in place of a pattern stamp.

### 2.3. Membrane Surface Geometry Characterization

Non-contact tapping-mode atomic force microscopy (AFM) was employed to observe the membrane surface morphology. Images were taken over 25 µm × 25 µm areas at a scan rate of 0.5 Hz using a Bioscope AFM (Bruker, Inc., Billerica, MA, USA) with silicon cantilever probe tips (MikroMasch, Inc., HQ:NSC16/AL BS, Watsonville, CA, USA).

### 2.4. Filtration Experiments

Feed solutions for all experiments were prepared by dispersing 200 mg/L silica nanoparticles (SiNPs) in DI water. Monodisperse SiNPs with a particle size of 70 ± 30 nm were prepared using the Stöber–Fink–Bohn method described elsewhere [25]. Briefly, a solution comprising ethanol (25 mL), ammonium hydroxide solution (5 mL), and DI water (1 mL) was stirred vigorously while 10 mL of TEOS was added dropwise to the solution. The mixture solution was stirred overnight at room temperature, sonicated and centrifuged to separate nanoparticles from solution. SiNPs were dried under vacuum (0.06 bar) at 100 °C for 1 h.

Data collection for threshold flux determination was performed using a custom filtration system designed to operate with constant permeate flux in the recycle mode to avoid changes in concentration of the feed solution. The filtration system details were described elsewhere [25]. Cross-flow velocity was held constant at 0.25 m s^−1^, and membrane samples were tested with the flow perpendicular to the patterns to reduce the rate of fouling relative to other orientations [31]. All experiments were conducted at 23 ± 1 °C. Prior to the filtration experiments, the feed solution was sonicated for 1 h, and membranes were preconditioned by operating the system with DI water for 15 min. Threshold flux values were measured for all six membrane types using the flux-stepping method. A starting permeate flux of 55 ± 5 L m^−2^ h^−1^ (LMH) was established, and transmembrane pressure (TMP) was adjusted to maintain constant flux for 10 min. Flux was increased stepwise in 20 LMH increments by adjusting the TMP. For each flux step, TMP again was adjusted to maintain constant flux for 10 min. TMP values ranged from 2.2 to 17.2 bar over the course of operation.

Constant-pressure flux decline experiments were conducted for all membrane types using an initial permeate flux of 140 ± 2 LMH, which was below the threshold flux for all samples. TMP was adjusted to have the same starting permeate flux for all samples. Cross-flow velocity was held constant at 0.25 m s^−1^, and the flow direction was perpendicular to the surface patterns. Flux data were collected every minute for patterned, pressed, and as-received membranes. Each filtration measurement was repeated three times.

### 2.5. Computational Fluid Dynamics Simulations

Multiple membrane models with line-and-groove surface patterns were built for analysis. Details of the developed model were explained elsewhere [22]. Table 2 summarizes the parameters of the four geometries (four stamps) that were studied. The velocity and pressure values are consistent with the experimental settings. The models were run on COMSOL Multiphysics 5.3.

Equations (1) and (2) are governing equations. Equation (1) is the Navier–Stokes equation that is used to describe the motion of the fluid, and Equation (2) is the continuity equation. Details of the modeling conditions were described elsewhere [22].

(1) $$\rho (\nabla \cdot u)u\;=\;-\nabla P+µ\nabla \cdot (\nabla u+\nabla u^{T}),$$

(2) ∇·u = 0,

## 3. Results and Discussion

### 3.1. Membrane Patterning

Table 1 shows the cross-sectional image and dimensions of each silicon stamp. Patterns consist of line-and-groove features with different spacing, from 150 to 300 nm for lines and 200 to 400 nm for grooves. The sample codes denote the percentage of the projected line surface area to total projected surface area of stamps. Pattern height was not considered as an independent parameter in this study due to the low thickness of the polyamide active layer. Precautions were made to avoid fracturing of the NF membrane active layer during patterning. The embossing pressure was set at 3.55 MPa to keep the local strain, defined by the height-to-pitch ratio of the pattern, below the cracking strain of nanoscale cross-linked polyamide films (14.04 ± 4.12% [32]), as Weinman and Husson [33] have suggested.

**Figure 1:**
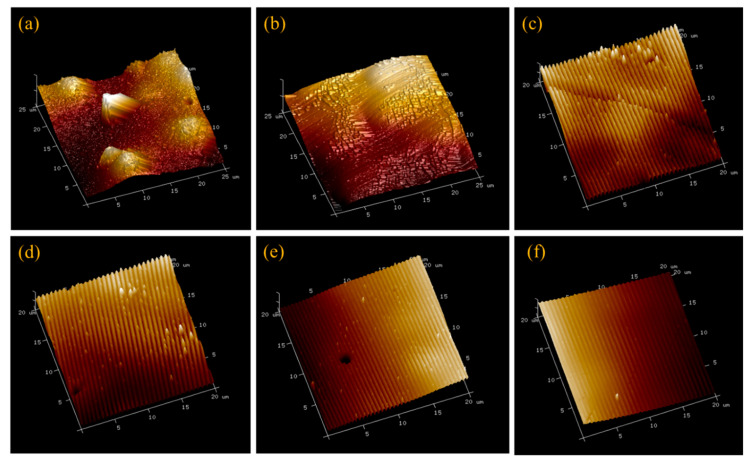
Representative AFM images of the six membrane types. (**a**) As-received membrane, (**b**) pressed membrane, (**c**) P27, (**d**) P31, (**e**) P50, and (**f**) P60. The scales are 25 μm × 25 μm × 600 nm for images (**a**,**b**) and 20 μm × 20 μm × 600 nm for images (**c**–**f**).

Figure 1 presents representative AFM images of all six membrane types. The groove depths and peak-to-peak distance for patterned membranes were determined by NanoScope Analysis 1.5 software and summarized along with local strain values in Table 3. While the average groove depth was limited to about 60 nm to avoid cracking of the active layer, the measured peak-to-peak distances are close to the stamp feature sizes, demonstrating successful replication of the stamp patterns on the membrane surfaces.

### 3.2. Threshold Flux Measurements

Colloidal fouling is a surface phenomenon that is affected by physical and chemical properties of the membrane surface [12] and hydrodynamics at the membrane–solution interface [25]. All membranes in this study had the same chemical properties, and cross-flow velocity and SiNP type and concentration were held constant. Thus, differences in fouling behavior can be attributed to differences in membrane surface morphologies due to patterning.

To assess the effect of different pattern geometries on fouling behavior, threshold flux values of as-received, pressed, and four types of patterned membranes were determined using constant flux cross-flow filtration experiments. We selected threshold flux as the test metric because of its importance to industrial practice. Operating just below threshold flux offers the possibility of achieving a continuous high flux while consuming less energy and decreasing the frequency of membrane cleaning [34]. A previously derived combined intermediate pore blocking and cake filtration model [30] was applied to the experimental data to determine threshold flux values quantitatively and in a systematized way. The time-dependent transmembrane pressure (TMP_t_) for the combined model is defined by Equation (3) [30]:

(3) $${\mathrm{TMP}}_{t}=\;\frac{{\mathrm{TMP}}_{0}\left(1+{\;K}_{c}\mathrm{Jt}\right)}{\left(\frac{1}{K_{i}}+\;\left(1-\frac{1}{K_{i}}\;\right)\exp\left(-K_{i}\mathrm{Bt}\right)\right)},$$

TMP_0_ is initial transmembrane pressure [N/m^2^], K_c_ is the cake filtration constant for cross-flow filtration [m^−1^], K_i_ is the intermediate pore blocking constant for cross-flow filtration, and B is the particle resuspension rate [s^−1^]. At the early stage of fouling, where the cake formation mechanism is absent, data can be fitted to the combined model with K_i_ equal to 1, and K_c_ values can be calculated. Details on this procedure are described elsewhere [25].

**Figure 2:**
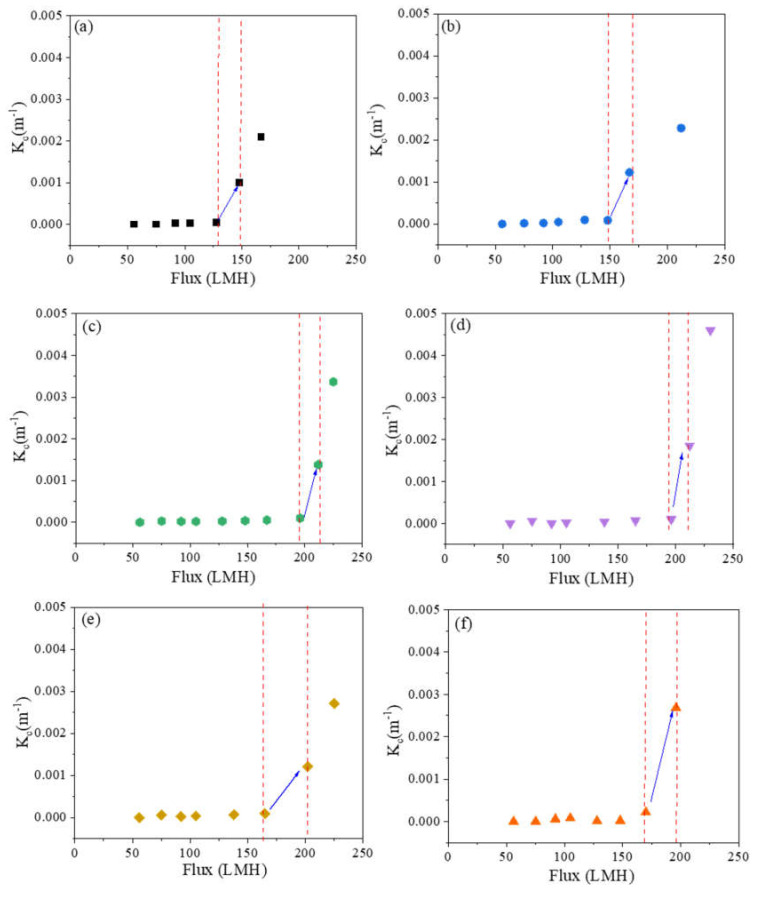
Dependence of the cake filtration constant on flux for (**a**) as-received, (**b**) pressed, (**c**) P27, (**d**) P31, (**e**) P50, and (**f**) P60 membranes.

**Figure 3:**
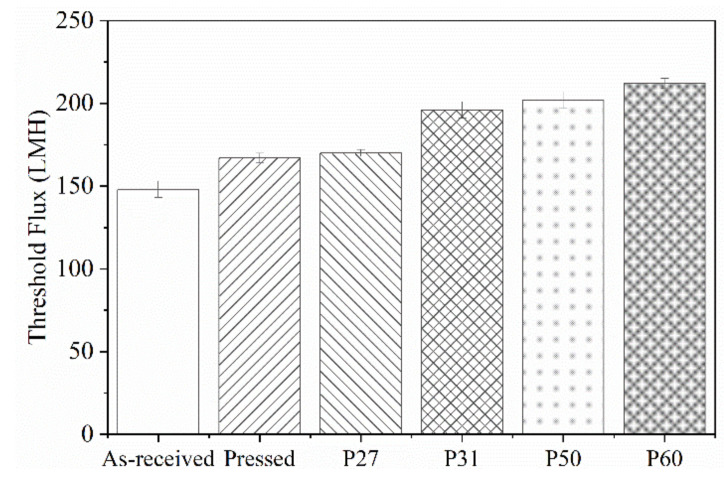
Threshold flux of as-received, pressed and patterned membranes. Error bars represent 95% confidence.

Figure 2 shows fitted values of K_c_ versus flux. The sharp rise in K_c_ shows the transition from intermediate pore blocking to cake filtration, which we use to define threshold flux. Figure 3 summarizes the threshold flux results, which depend strongly on the membrane surface structure. The threshold flux of all patterned membranes is higher than the as-received and pressed membranes. For patterned membranes, more fouling is observed when there is a low fraction of projected line surface area. Pressed membranes had higher threshold flux than as-received membranes. This result agrees with previous studies that showed how decreasing the nanoscale surface roughness of a membrane by pressing the membrane can decrease fouling [1,35]. The effectiveness of the “ordered roughness” on patterned membranes for increasing threshold flux could be related to increased membrane surface area and different hydrodynamic properties at the membrane–solution interface [26,33]. Patterning increases the overall surface area of the membrane. Since flux is calculated based on projected membrane area, increasing the overall area leads to higher flux values. It is known that the groove region of patterned membranes is more disposed to fouling as a result of unequal feed flow distribution [26], lower shear stress in groove regions compared to line regions [36], and lower hydrodynamic drag force compared to attractive interactions between foulant and membrane [37]. Therefore, more severe fouling may be expected for membranes with higher fractions of groove surface area, which agrees with the experimental results of this study; threshold flux increased in the following order: as-received < pressed < P27 < P31 < P50 < P60. To analyze the relationship between threshold flux and pattern geometry quantitatively, we defined the pattern fraction ratio (PFR) as the quotient of line width (b) and groove width (a).

(4) $$\mathrm{PFR}=\frac{b}{a},$$

**Figure 4:**
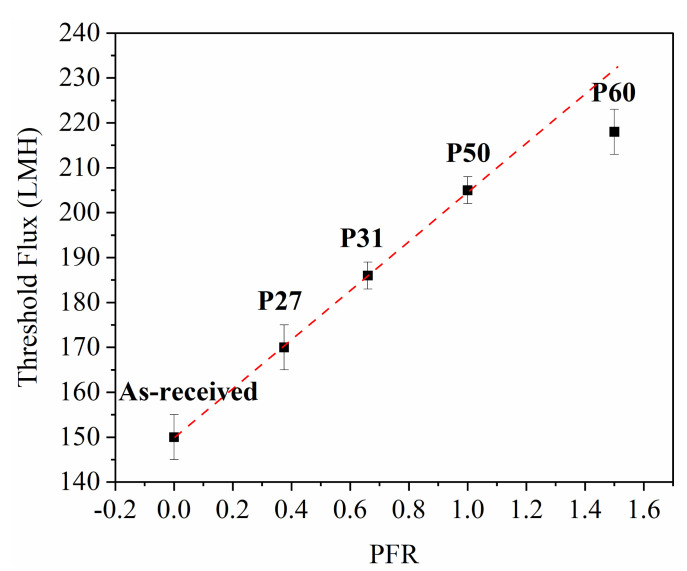
Relationship between threshold flux and pattern fraction ratio. Error bars represent standard deviation among three threshold flux measurements.

Figure 4 shows that there is a linear correlation between threshold flux and the PFR, with the exception of P60 at the highest PFR value.

**Figure 5:**
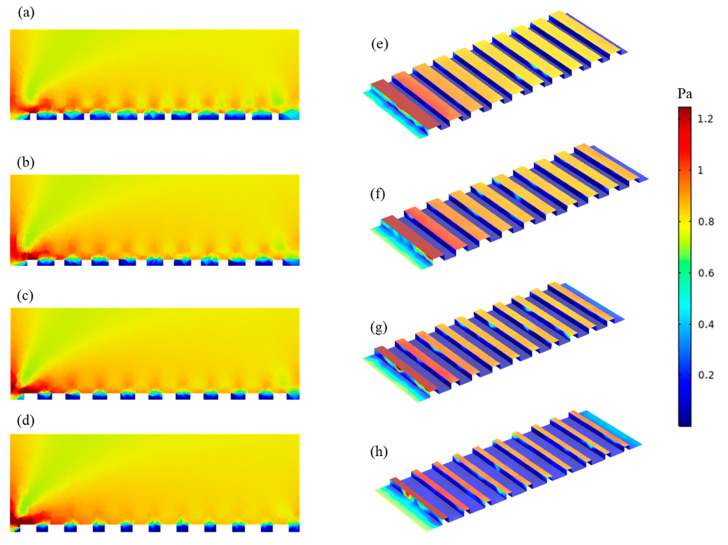
Shear stress profile along the membrane: (**a**–**d**) 2D ((**a**) P27, (**b**) P31, (**c**) P50 and (**d**) P60); (**e**–**h**) 3D ((**e**) P27, (**f**) P31, (**g**) P50 and (**h**) P60). Red color indicates a higher shear stress and blue indicates a lower shear stress.

**Figure 6:**
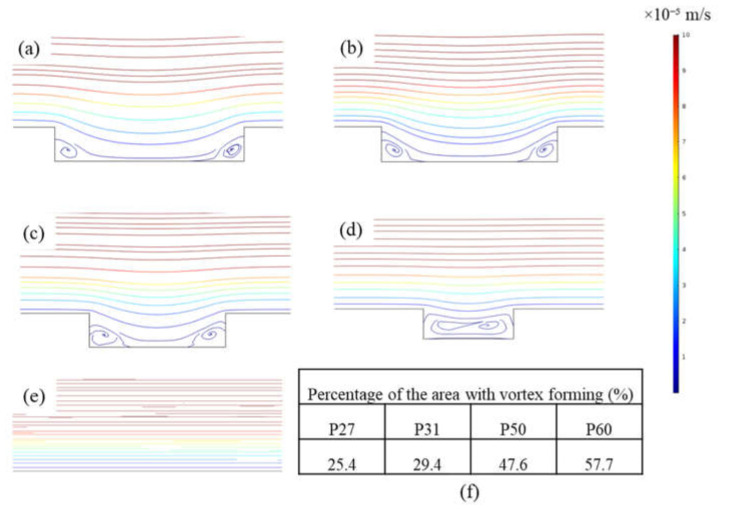
Streamline profiles for (**a**) P27, (**b**) P31, (**c**) P50, (**d**) P60, and (**e**) flat membrane; and (**f**) percentage of the area with vortex forming for patterned membranes. Red color indicates a higher velocity and blue indicates a lower velocity.

To better understand the basis for this result, CFD simulations were carried out to analyze shear stresses and local flow behavior close to the membrane surfaces. Figure 5 shows the shear stress profiles of patterned membranes. Figures on the left show the 2D profiles on and near the membrane surface including part of the channel, and those on the right show the 3D profiles of the shear stress distribution on the membrane surface for all geometries. Higher shear stresses develop on the peaks and lower shear stresses are found in the valleys. Values decrease along the length of the channel. Simulations indicate that the P60 pattern has the highest average shear stress (0.46 Pa), while the P27 pattern has the lowest average shear stress (0.28 Pa). This finding agrees with the experimental results that show threshold flux is highest for P60 and lowest for P27 among patterned membranes; however, the differences in shear stress are not large. Therefore, streamline profiles for all pattern geometries were studied. Figure 6 shows streamline profiles of flat and patterned membranes. Vortices developed in all valley regions near the patterned membrane surfaces. Vortex formation separates bulk flow from flow inside the grooves, and the chance for particle transfer from grooves to bulk flow decreases, leading to increased fouling. Choi et al. [12] referred to this phenomenon as vortex-induced shielding. In addition, the velocity is lower inside the grooves than in the rest of the channel, which contributes to the enhanced fouling inside of grooves. Vortex formation was more complicated for the P27, P31, P50 patterns, as shown in Figure 5. Two small vortices developed, which may explain the higher rate of colloidal fouling. The area percentage of the vortex-forming region was calculated as Choi et al. [24] suggested. By increasing the groove width, the area percentage increases and therefore, the effect of vortex-induced shielding is higher.

CFD simulation results explained the cause for the increasing trend of threshold flux with the PFR. As the PFR increases, the percentage of vortex-forming area increases, and vortex-induced shielding increases. In Figure 5, bulk flow streamlines, which have the highest velocity, are separated from vortices that have a lower velocity by a transitional region. A high thickness of transitional region between bulk and vortex flow can decrease the chance of particle skipping from vortex flow to bulk flow [20]. Therefore, fouling rate increases and threshold flux decreases. Our study suggests that the PFR should be higher than 1 to maximize threshold flux values of patterned membranes.

Noteworthy is that the CFD simulations were carried out without the introduction of particles. Thus, given the correlation between the PFR and the percentage of vortex-forming area determined by the particle-free CFD simulations, we expect the findings to be generalized to other particle sizes, provided that the particles are smaller than the groove width. As discussed by Maruf et al. [38], the highest critical (threshold) flux is found for colloidal particles closest in size to the groove width. Particles with diameters below and above this width can be expected to yield lower threshold flux.

As discussed earlier, patterning increases overall membrane surface area. The surface area increased in the following order: P60 < P27 < P50 < P31. Our results show that there is no correlation between the increase in threshold flux and increase in surface area. This finding further suggests that the hydrodynamic properties at the membrane–solution interface have greater influence on fouling than membrane surface area.

### 3.3. Flux Decline Measurements

**Figure 7:**
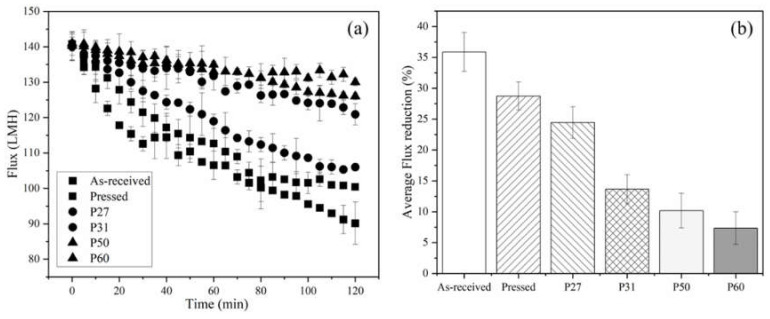
(**a**) Flux decline data for all six membrane types. (**b**) Average flux reduction over the 2 h test runs for all membranes. Temperature was 22–23 °C, cross-flow velocity was 0.25 m s^−1^, and tested membrane area was 6.37 cm^2^. The initial flux was 140 ± 2 LMH for all samples. The error bars represent standard deviation among three filtration tests.

Figure 7a shows flux versus time data collected for filtration of 200 mg/L SiNPs with all membrane types. To allow direct comparison of results, the initial permeate flux for all membranes was set at 140 ± 2 LMH, which is 10 LMH lower than lowest threshold flux value of 150 LMH for as-received membrane. Figure 7b shows that as-received membranes experienced the largest decrease in the flux (35%) over the 2 h test run. Pressing the membrane improved the fouling resistance due to the decrease in intrinsic membrane surface roughness, as Weinman and Husson [33] showed. Lower surface roughness decreases the rate of colloidal particles attachment to the membrane by providing less contact surface area between the membrane surface and particles [13]. In all cases, patterning lessened the flux decline more than pressing alone. Patterning changes the hydrodynamic properties at the membrane–solution interface, as discussed above. Overall, flux decline results aligned with threshold flux measurements. By measuring and operating below the threshold flux, less fouling occurs, and the frequency of membrane cleaning can be decreased.

## 4. Conclusions

This study revealed the effect of line-and-groove pattern geometry on threshold flux for filtration of colloidal nanoparticle suspensions through patterned nanofiltration membranes. Experimental work combined with CFD simulations showed that increasing the pattern ratio fraction leads to higher threshold flux, which is important for increasing the volumetric productivity of water treatment systems while maintaining low rates of fouling. Modeling provided insights into the fouling mechanism of colloidal particles on these membranes, and the transition from intermediate pore blocking to cake filtration was used to calculate threshold flux quantitatively in a systematic way. The results of this study can be extended to investigate the effect of pattern geometry for other feature types such as herringbone, pyramid, and biomimetic patterns.

## Figures and Tables

**Table 1:** Cross-sectional image and dimensions of the line-and-groove pattern for each stamp.

Pattern ID	Pattern Geometry
P27	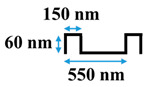
P31	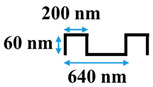
P50	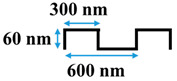
P60	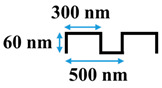

**Table 2:** Parameters of the geometries.

Pattern ID	Pattern Depth, *h* (nm)	Groove Size, *d* (nm)	Line Size, *l* (nm)	Cycle Length, *W = l + d* (nm)
P27	60	400	150	550
P31	60	440	200	640
P50	60	300	300	600
P60	60	200	300	500

**Table 3:** Feature sizes on patterned membranes measured by AFM.

Pattern ID	Averaged Pattern Depth (nm)	Averaged Peak-to-Peak Distance (nm)	Local Strain (%)
P27	58 ± 6	530 ± 20	10.1 ± 3.2
P31	60 ± 8	600 ± 30	10.3 ± 5.6
P50	66 ± 7	680 ± 70	10.9 ± 5.4
P60	65 ± 6	510 ± 20	10.8 ± 6.2
